# Tumor location and type affect local recurrence and joint damage in tenosynovial giant cell tumor: a multi-center study

**DOI:** 10.1038/s41598-021-96795-6

**Published:** 2021-08-30

**Authors:** Takehiro Ota, Yoshihiro Nishida, Kunihiro Ikuta, Satoshi Tsukushi, Kenji Yamada, Eiji Kozawa, Hiroshi Urakawa, Shiro Imagama

**Affiliations:** 1 Department of Orthopaedic Surgery, Nagoya University Graduate School of Medicine, Nagoya, Aichi Japan; 2 Department of Rehabilitation Medicine, Nagoya University Hospital, 65 Tsurumai, Showa, Nagoya, Aichi 466-8550 Japan; 3 Department of Orthopaedic Surgery, Aichi Cancer Center Hospital, Nagoya, Aichi Japan; 4 Department of Orthopaedic Surgery, Okazaki City Hospital, Okazaki, Aichi Japan; 5 Department of Orthopaedic Surgery, Nagoya Memorial Hospital, Nagoya, Aichi Japan

**Keywords:** Medical research, Oncology, Risk factors

## Abstract

Osteochondral destruction and a high recurrence rate after surgery are major concerns that make difficult the treatment course of tenosynovial giant cell tumor. The aims of this study were to elucidate rates of postoperative local recurrence and osteochondral destruction, as correlated with various demographic factors. Eighty surgically treated patients with intra-articular tumors (knee: 49, ankle and foot: 12, hip: 10, others: 9) were included in this study. Factors including age, disease type (diffuse/localized), location, existence of osteochondral destruction were correlated with local recurrence or development/progression of osteochondral destruction. The 5-year local recurrence free survival rate was 71.4%. Diffuse type (n = 59, localized: n = 21) (P = 0.023) and knee location (P = 0.002) were independent risk factors for local recurrence. Diffuse type (P = 0.009) was a significant risk factor, and knee location (P = 0.001) was a negative factor for osteochondral destruction at the initial examination. Progression of osteochondral destruction was observed more often in cases with local recurrence (P = 0.040) and findings of osteochondral destruction at the initial examination (P = 0.029). Diffuse type is a factor that should be noted for both local recurrence and osteochondral destruction, while local recurrence occurs but osteochondral destruction is less observed in the knee.

## Introduction

Tenosynovial giant cell tumor (TSGCT), which has also been called pigmented villonodular synovitis (PVNS), is a rare disease characterized by proliferation of synovial tissue arising usually in larger joints, such as knees or hips^1^. The annual incidence of TSGCT was estimated to be 1.8 patients per million population in the United States^2^, and more recently 4 per million by Mastboom et al.^3^. TSGCT occurs predominantly in the third to fifth decades, and rarely in children or the elderly^4^. The pathogenesis of TSGCT, namely whether neoplastic or reactive proliferation is implicated, has been discussed^5,6^. Recent findings showing that TSGCT is a clonal chromosomal abnormality suggest TSGCT to be a neoplastic disorder^7,8^. Based on these reports, the name of TSGCT proposed by the World Health Organization has become widely used and classified as localized type and diffuse type, so-called PVNS^9^.

Diffuse type is known to have a high recurrence rate after surgery^10^ and occasionally causes osteochondral destruction^11,12^. Clinical concerns of diffuse type after treatment include local recurrence after surgery and progression of osteochondral destruction. Local recurrence is frequently observed in this disease, particularly in patients with knee involvement^13^. On the other hand, patients with hip and ankle involvement are prone to the development and/or progression of osteochondral destruction^12,14–16^. Simple en bloc resection has been performed for the localized type, with thorough excision of synovial tumorous tissues having long been the standard therapy for the diffuse type. Arthroplasty, arthrodesis, and artificial joint replacement have been performed in patients with severe joint destruction, particularly in the diffuse type^17^. The multiple institutions participating in the present study performed an identical surgical treatment until recently as described above. However, in the past decade, the present authors have employed open total excision for posterior tumors and arthroscopic excision for anterior tumors in knee cases with both anterior and posterior tumor involvement to reduce the operative stress and obtain a favorable range of motion of the knee.

Main concerns of TSGCT treatment are local recurrence and progression of osteochondral destruction, because they occasionally cause intolerable pain, and impair the function of involved joints. Considering that TSGCT is a benign neoplasm, treatment should focus on not only reduction of the local recurrence rate and oncological outcome, but also preservation of the function of the involved joint. Several reports have described the development of bone destruction in patients with TSGCT^11,18–20^. However, previous studies did not focus on the progression of bone destruction, or the occurrence or progression of osteoarthritic change (bone change in addition to narrowing of the joint space) of the involved joint. Our previous study, which was a single institutional study, reported that local recurrence and subsequent re-operation significantly correlated with progression of osteoarthritic change, although these results did not reach significance regarding bone destruction in cases with knee lesions, possibly due to the small number of such cases^21^. The previous study included cases without surgery, and investigated the correlating factors with univariate analyses.

This prompted us to collect only surgically treated cases from affiliated soft tissue tumor centers, and analyze the local recurrence rate, occurrence/progression of osteochondral destruction and associated factors with multivariate analyses in TSGCT. The relationship between operative procedure including arthroscopic excision and recurrence rate or osteochondral destruction was also investigated.

## Methods

**Figure 1:**
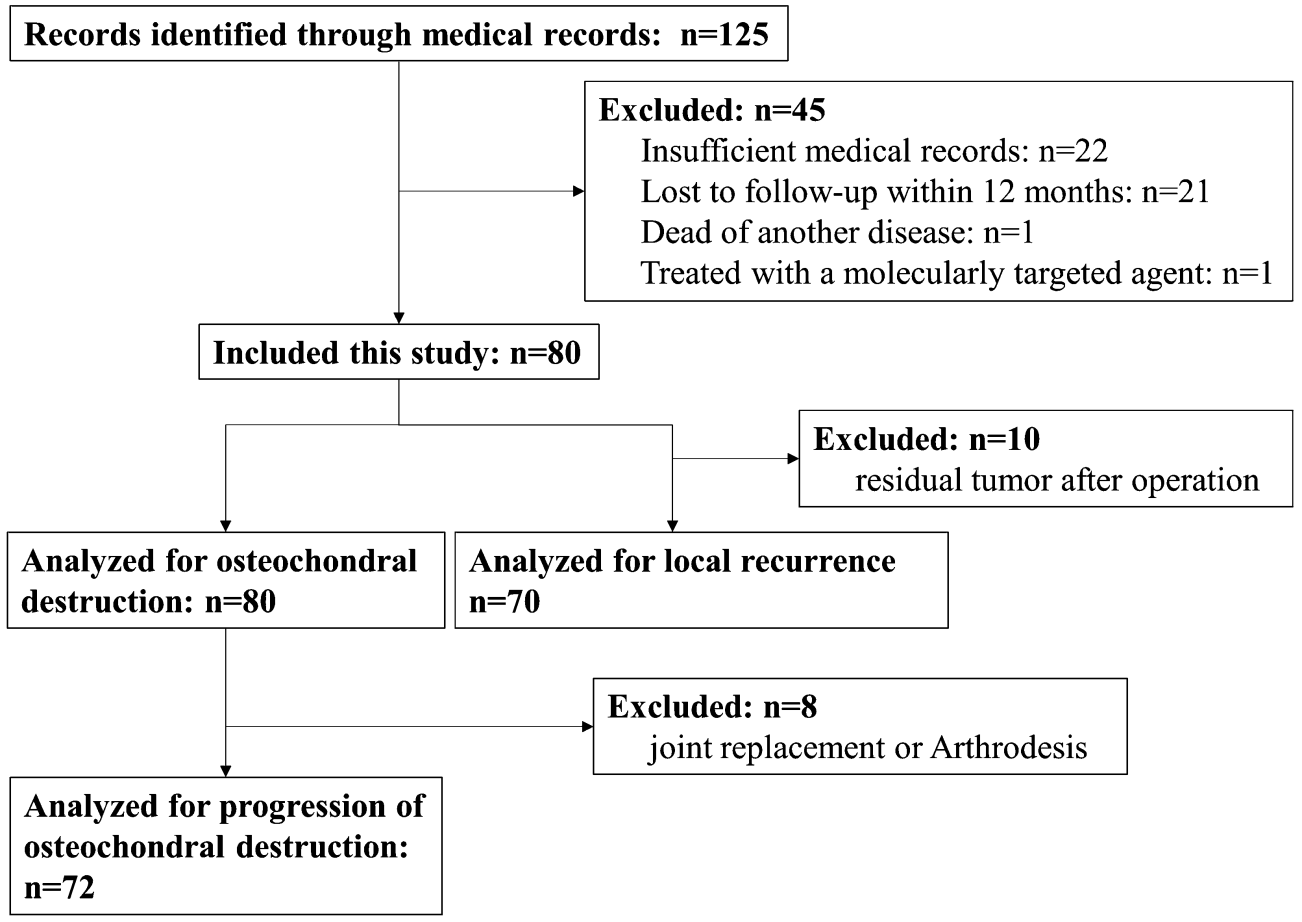
The STROBE flow diagram of the study.

We retrospectively reviewed the medical records of 125 patients who had a definitive histological diagnosis of TSGCT made by experienced pathologists and who had undergone surgery in our institutions from 1990 to 2012. Although cases with tumors involving intra-articular spaces were included, those limited to extra-articular regions were excluded from this study. We collected information including age, sex, involved joints, existence or progression of osteochondral destruction (including bone destruction and osteoarthritic change), surgical procedures, local recurrence, and subsequent treatment including repeated surgery after relapse. Twenty-two cases with insufficient medical records, 21 cases lost to follow-up within 12 months, one case dead of another disease, and one case treated with a molecularly targeted agent before surgery were excluded. As a result, 80 cases were included in this study. The STROBE flow diagram of the study is presented in Fig. 1. Our institutional review board approved this study (approval number: 5259). In this approval, the need for informed consent was waived by Nagoya University because of the retrospective design of the study based on anonymous data. For other facilities, IRB was waived by communicating the research contents to the director because the study was a retrospective one, and did not collect new samples. This research was conducted in accordance with the principles set out in the Declaration of Helsinki.

### Radiological examination

All patients were examined by plain anteroposterior and lateral radiography and magnetic resonance imaging (MRI) of affected joints at the time of the initial referral.

The distinction between localized and diffuse types was made based on the MRI images. A single circumscribed lesion in MRI T2 images was defined as localized type, and other multiple and/or infiltrating lesions as diffuse type. In cases in which the differentiation of these lesions was difficult, two observers (T.O. and K. I.; orthopedic oncologists) made these decisions after careful discussion.

Presence of bone destruction was assessed with plain radiographs and/or computed tomography (CT) images, and lesions > 5 mm in diameter were evaluated as positive because 5 mm is considered the detection limit by plain radiography. Progression of destruction was defined as extension of bone destruction by more than 5 mm. Osteoarthritic change which reflects cartilage degeneration and osteophyte formation was evaluated using the Kellgren–Lawrence (K–L) scale^22^, a 5-level OA severity score (0 indicating normal; 1 doubtful; 2 mild or minimal; 3 moderate; and 4 severe) based on a pictorial guide depicting the degree of osteophyte formation, joint space narrowing, sclerosis, and joint deformity. Progression of osteoarthritic change was defined as an increase in the K-L score on plain XP examination during the clinical course. OA change was defined as K-L grade equal or more than II. Two observers (T. O. and K. I.) evaluated bone destruction and osteoarthritic change separately. Osteochondral destruction was defined as the presence of bone destruction and/or osteoarthritic change. Kappa statistics were used to determine the interobserver variability. Kappa coefficient showed good agreement for observers evaluating the osteochondral destruction (initial: 0.856, progression: 0.845). If any discrepancy was present in the evaluation, they discussed it together until reaching a consensus.

Local recurrence was evaluated using MRI every 3 to 6 months postoperatively. When MRI showed a new lesion larger than 1 cm with TSGCT-like signal intensity during the postoperative course where the surgical team should have removed the macroscopic tumor completely, and the lesion did not decrease in size in the follow-up MRI image, it was considered to be a local recurrence.

### Demographics

Forty-nine patients were females and 31 were male. Fifty-nine cases had diffuse and 21 had localized disease. Forty-nine cases showed knee involvement, 12 of ankle and foot, 10 of hip, 4 each of elbow and wrist, and one of shoulder. Mean age was 39.0 years (5–80) and mean follow-up was 66 months (12–226 months). Twenty-six cases had osteochondral change at the initial examination. (Table 1).

**Table 1:** Demographic data of 80 cases.

Variables	N
**Sex**	
Male	31
Female	49
**Age. years (range)**	39 (5–80)
**Disease type**	
Localized	21
Diffuse	59
Localization	Cases (localized/diffuse)
Shoulder	1 (0/1)
Elbow	4 (2/2)
wrist	4 (1/3)
Hip	10 (0/10)
Knee	49 (14/35)
Ankle and foot	12 (4/8)
**Local recurrence**	
Yes	22
No	48
Residual tumor	10
**Osteochondral change (initial)**	
Yes	26
No	54
**Osteochondral destruction (progression)**	
Yes	22
No	50
Evaluation impossible	8
**Follow-up, months**	
Median	66 (12–226)

### Treatment

All 80 patients underwent surgical excision of tumors. The surgical treatment modality for diffuse type TSGCT was open surgery with sufficient tumor excision. Diffuse type tumors arising in knees were also generally treated with open anterior and/or posterior total tumor excision. Thirteen recent cases of diffuse TSGCT, which could be treated arthroscopically, were treated with arthroscopic synovectomy for anterior tumors, thereby probably facilitating preservation of knee function^23^. Arthroscopic surgery was performed by knee surgeons with more than 15 years’ experience of arthroscopy. Posterior tumors received open excision. However, some of the asymptomatic posterior tumors remained untouched based on detailed discussion between patients and physicians. Three cases of hip and 1 case of knee were treated with joint replacement, 2 cases of ankle, 1 case of wrist, and 1 case of knee were treated with arthrodesis after tumor removal. On radiological follow-up examination after surgery, a residual tumor was detected in ten patients (6 cases; knee, 4; ankle). In 9 of 10 cases, we intentionally planned incomplete tumor removal in order to preserve involved joint function. These 10 cases were included in the analyses for osteochondral destruction, but excluded from those for local recurrence. In the localized type of TSGCT, the tumor was simply excised en bloc. No patients received either radiotherapy or chemotherapy for TSGCT.

The patients and physicians determined the treatment strategy together in cases with local recurrence or residual tumor.

### Statistical analysis

Mann–Whitney *U* test was used to assess continuous variables including age as an influencing factor for local recurrence or osteochondral destruction. Fisher's exact test or Pearson's chi-square test was used to assess whether categorical variables (sex, disease type, involved joints) affect local recurrence or existence or progression of osteochondral destruction. Kaplan–Meier method was used to determine the local recurrence free survival rate (LRFS). Log-rank test and Cox regression analysis were used to evaluate the difference in LRFS. Logistic regression analysis was used to evaluate factors affecting osteochondral change at the initial examination and progression of osteochondral destruction. P values < 0.05 were considered as statistically significant. Statistical analysis was performed using SPSS software, version 27 for Windows (IBM Inc, NY, USA).

### Ethics approval and consent to participate

This study was approved by Institutional Review Board of Nagoya University Graduate School of Medicine (Approval Number: 5259).

## Results

### Local recurrence

**Figure 2:**
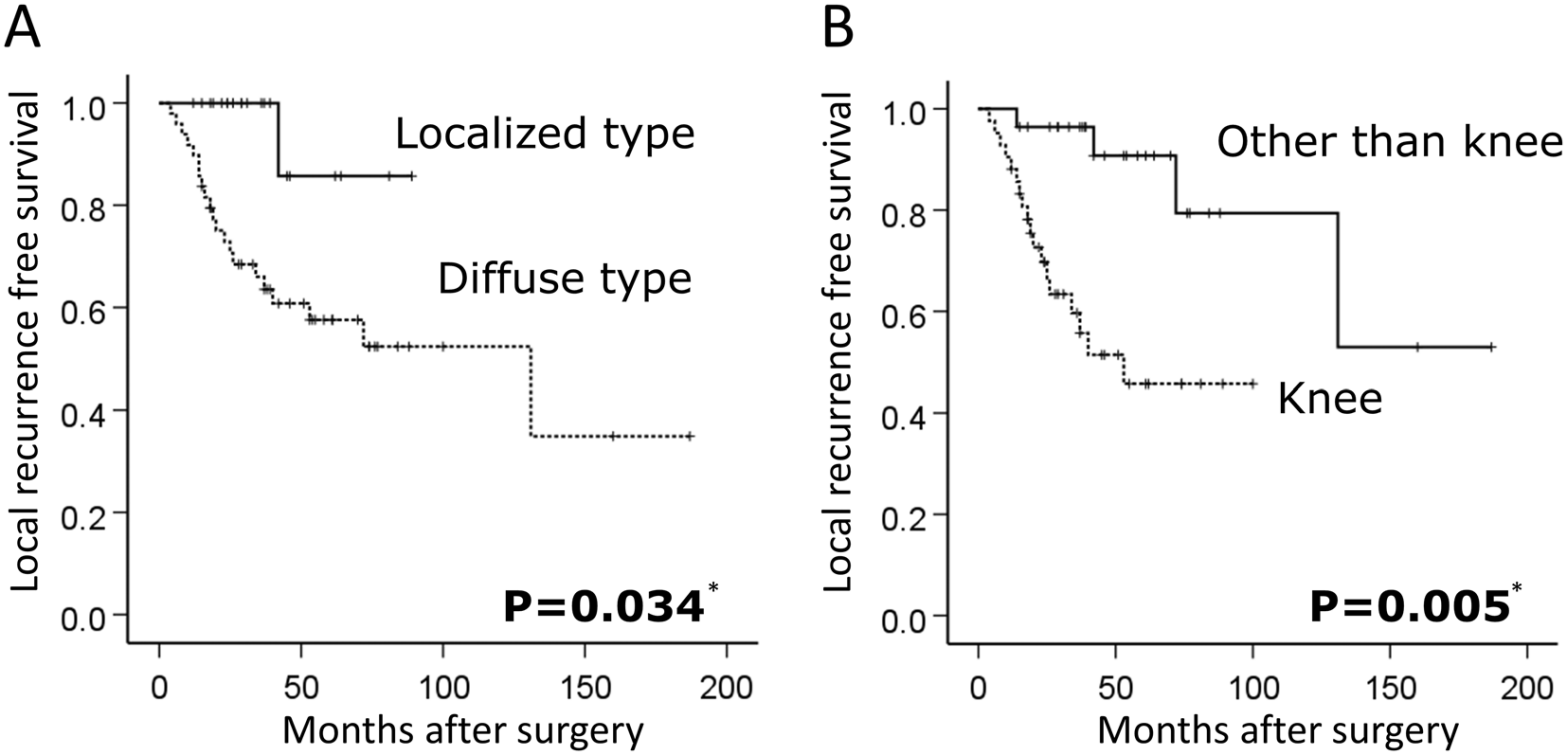
Local recurrence free survival. Local recurrence free survival was determined by Kaplan–Meier method. (**A**) The difference between the local recurrence-free survival rates of each group (localized vs diffuse) was statistically significant (P = 0.034). (**B**) Knee location was a significant risk factor for local recurrence (P = 0.005). *LFRS* Local recurrence free survival.

Of the 80 patients, 10 who had residual tumor after initial surgery were excluded. Local recurrence occurred in 22 (28%) cases and the mean time to local recurrence was 29 months (4–131). Twenty-one (95%) of 22 recurrent cases were diffuse type, and 18 (82%) of 22 were knee cases. Remaining 4 cases with local recurrence were ankle locations. There was no recurrence in the hip joint cases. The 5-year LRFS was 71.4%. 5-year LRFS for localized and diffuse tumors were 95.2% and 61.2%, respectively (P = 0.034, Fig. 2A). 5-year LRFS for knees and other joints were 57.1% and 92.8%, respectively (P = 0.005, Fig. 2B). A Cox regression analysis revealed that diffuse type (HR = 10.388, CI 1.391–77.583, P = 0.023) and knee lesions (HR = 7.157, CI 2.081–24.607, P = 0.002) were independent risk factors for local recurrence (Table 2).

**Table 2:** Cox regression analysis of Local recurrence free survival.

Variables	Univariate		Multivariate	
	P-value	HR (95% CI)	P-value	HR (95% CI)
Diffuse type	0.04	8.197 (1.095–62.49)	0.023	10.39 (1.391–77.58)
Knee lesion	0.004	5.941 (1.738–20.30)	0.002	7.157 (2.081–24.61)
Arthroscopic surgery	0.018	3.227 (1.225–8.502)	0.355	1.662 (0.567–4.876)
Osteochondral change	0.202	0.517 (0.188–1.425)		

*HR* hazard ratio, *CI* confidence interval.

Subgroup analysis of knee cases revealed that only diffuse type was a significant risk factor for local recurrence in both univariate (HR = 9.803, P = 0.026) and multivariate analyses (HR = 9.434, P = 0.033). Local recurrence occurred in 6 (46%) of 13 cases that underwent arthroscopic assisted surgery, and 12 (33%) of 36 cases open surgery, with this difference not significant (P = 0.411).

Re-operation was performed in eight (36%) of 22 cases with local recurrence. Re-operation was performed in all of four patients with relapse in the ankle due to issues such as pain and/or dysfunction.

### Osteochondral destruction at the initial examination

Evaluation of plain X-P and/or CT images at the time of the initial examination revealed osteochondral destruction in 26 (33%) of 80 patients. Osteochondral destruction was observed in 9 (18%) of 49 cases with knee lesion, in contrast to 9 (90%) of 10 cases with hip location, 2 (50%) of 4 with elbow, 2 (50%) of 4 with wrist, and 4 (33%) of 12 with foot and ankle. Twenty-five of 26 cases with osteochondral destruction at the time of the initial examination showed the diffuse type. Univariate analysis showed diffuse type (P = 0.002) and non-knee lesion (P = 0.001) to be significant risk factors for osteochondral destruction at the initial examination. Multivariate analysis also revealed diffuse type (OR = 17. 717, CI 2.068–151.765, P = 0.009) and knee location (OR = 0.159, CI 0.052–0.483, P = 0.001) to be independent negative and positive risk factors, respectively, of osteochondral destruction at the initial examination. (Table 3). Subgroup analysis of knee cases indicated that the only risk factor for osteochondral destruction at the initial examination was diffuse type (p = 0.045).

**Table 3:** Factors influencing osteochondral change at initial examination.

Variables	Osteochondral change		P-value (univariate)	OR (95% CI)	P-value*** (multivariate)
	No	Yes			
**Sex**			0.598*		
Male	22	9			
Female	32	17			
**Age, years**	38.0	41.3	0.356**		
**Disease type**			0.002*	17.717 (2.068–151.765)	0.009
Diffuse	34	25			
Localized	20	1			
**Site**			0.001*	0.159 (0.052–0.483)	0.001
Knee	40	9			
Others	14	17			

*OR* odds ratio, *CI* confidence interval.

*Fisher's exact test and Pearson chi-square test.

**Mann–Whitney U test.

***Logistic regression analysis.

### Progression of osteochondral destruction

**Figure 3:**
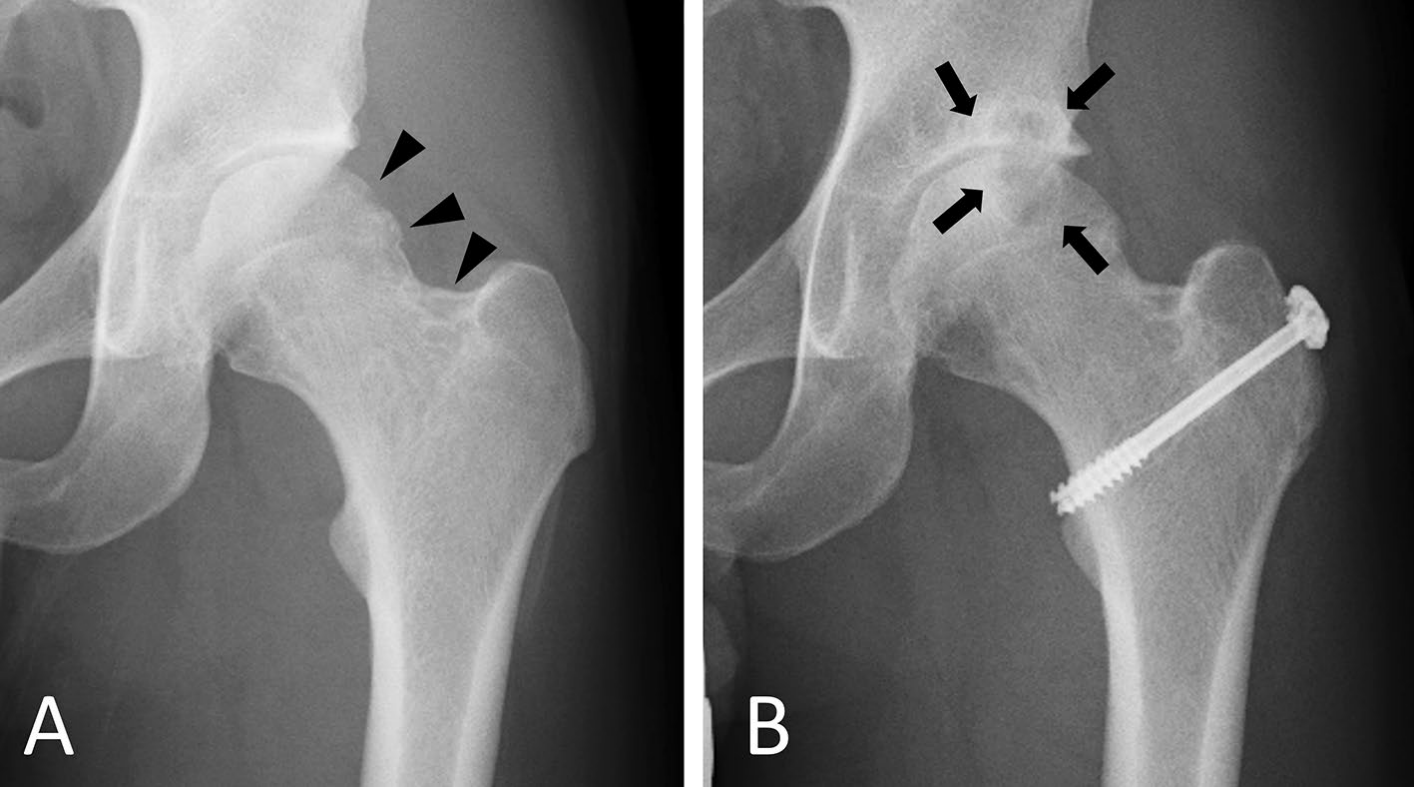
Osteochondral lesion in hip joint. (**A**) A 27-year-old men with hip TSGCT. An anterior–posterior radiograph showed osteolytic lesion in left femoral head and neck region (arrowheads), however there is no obvious osteoarthritic change at initial examination. (**B**) Three years after tumor resection. After surgery for TSGCT, osteoarthritic change occurred (arrows).

We excluded eight patients who were treated with joint replacement or arthrodesis from the analyses for progression of osteochondral destruction. Progression of osteochondral destruction during follow-up was observed in 22 (31%) of 72 cases (Fig. 3). Fourteen cases of knee lesions, 5 of hip, 2 of ankle, and 1 of wrist showed progression. All 22 cases with progression of osteochondral destruction were diffuse type. Eleven (50%) of 22 cases had an osteochondral change at the initial examination. Univariate analysis revealed that diffuse type (P < 0.001), local recurrence (P = 0.012), and osteochondral change at the initial examination (P = 0.018) were significant factors for the progression of osteochondral destruction (Table 4). Multivariate analysis revealed that local recurrence (OR = 3.747, CI 1.065–13.180, P = 0.040) and osteochondral destruction at the initial examination (OR = 4.084, CI 1.157–14.411, P = 0.029) were independent risk factors for progression of osteochondral destruction (Table 4). Subgroup analysis of knee cases revealed that diffuse type (P = 0.004) and local recurrence (P = 0.033) were significant risk factors with univariate analysis. Local recurrence (OR = 5.039, P = 0.042) was the sole significant factor identified in the multivariate analysis.

**Table 4:** Factors influencing osteochondral destruction during follow-up.

Variables	Osteochondral destruction		P-value (univariate)	OR (95% CI)	P-value*** (multivariate)
	No	Yes			
**Sex**			0.475*		
Male	22	7			
Female	28	15			
**Age, years**	37.6	37.9	0.952**		
**Disease type**			< 0.001*	N.A	
Diffuse	29	22			
Localized	21	0			
**Site**			0.846*	2.805 (0.561–14.040)	0.209
Knee	33	14			
Others	17	8			
**Local recurrence**			0.012*	3.747 (1.065–13.180)	0.040
No	34	8			
Yes	10	10			
**Repeated surgery**			0.082*		
No	45	16			
Yes	7	7			
**Osteochondral change (initial)**			0.018*	4.084 (1.157–14.411)	0.029
No	39	11			
Yes	11	11			

*OR*  odds ratio, *CI*  confidence interval, *N.A.* not available.

*Fisher's exact test and Pearson chi-square test.

**Mann–Whitney U test.

***Logistic regression analysis.

### Relationship between progression of osteochondral destruction and tumor location

The progression rate of osteochondral destruction varied among tumor locations. In the analyses of knee lesions, two cases were excluded because of arthrodesis and total knee arthroplasty. In 14 patients (14/47, 30%) with progression of osteochondral destruction, arthroscopic assisted surgery was performed only in 1 case, and open surgery in the remaining 13 cases at the initial surgery (P = 0.072). In addition, eight of the 14 cases with osteochondral progression were recurrent ones. As a result, total knee arthroplasty was performed in 4 of the 8 cases, and knee contractures occurred in the remaining four cases to a varying degree. Residual tumor was observed after initial surgery in six knee cases (13%). In three of these cases, the posterior lesions were intentionally left behind at the initial surgery in consideration of the associated surgical invasiveness. None of the 6 cases with residual tumor required re-operation. Osteochondral destruction slowly progressed in 2 of the 6 cases.

Arthrodesis was performed in two of the 12 cases of ankle involvement at the time of the initial surgery. Of the 10 cases evaluated, osteochondral destruction progressed in 3 cases. Two of the 3 cases were recurrent cases, and extended tumor resection including the surrounding joint capsule was performed in the other case.

Three of 10 cases of hip involvement were excluded because total hip arthroplasty was performed at the time of the initial surgery. Three of the remaining 7 cases received tumor excision with surgical dislocation of the involved hip. Osteochondral destruction progressed in 5 of the 7 cases evaluated (71%), and total hip arthroplasty was performed in one.

## Discussion

Although several studies have identified risk factors for local recurrence^24,25^ in TSGCT, none have analyzed possible risk factors for osteochondral destruction and progression in combination with those for local recurrence. Given that TSGCT is a benign neoplasm^9^, the primary endpoint of treatment should be to maintain the function of involved joints. Surgery to reduce the recurrence rate in TSGCT occasionally causes morbidity of involved joints. This makes it important to analyze factors influencing local recurrence and osteochondral destruction together.

Previous studies reported that TSGCT has a high local recurrence rate of 16–30%^10,11,24^, with the rate high in the diffuse type^10,11^, and in knee lesions^11,24^. These results are consistent with those in the present study, and the local recurrence free survival rate in the present study was also comparable with that noted in past reports^10,11^, indicating that the quality of treatment in our institutions is roughly equal to that reported elsewhere. The treatment modality for primary/recurrent TSGCT has been surgical excision of tumors, but is now being partially changed to watchful observation, particularly for recurrent tumors if morbidity is expected with surgical treatment in our institutions. Recently, arthroscopic surgery has been applied to TSGCT surgery, particularly for knee involvement^26–29^. Several previous reports described comparable results to open surgery regarding local recurrence^27,28^, whereas others described inferior results^26,29^. Most recent studies also reported conflicting results regarding the clinical outcome of arthroscopic surgery (Table 5)^30,31^. Although the local recurrence rate was higher in arthroscopic surgery than that in open synovectomy in the present study, there was no significant difference probably due to the small numbers of cases, shorter follow-up period, and possible selection bias (easy to approach cases) in cases treated with arthroscopic surgery. Most of the previous studies describing arthroscopic surgery reported the results of local recurrence, but not the functional results, which seem to be superior with arthroscopic surgery as compared with open synovectomy. A recent study reported postoperative function, but unfortunately only 6 of 206 cases had undergone arthroscopic surgery (Table 5)^32^. Focusing on the results of local recurrence, arthroscopic surgery may have a disadvantage compared to open synovectomy. However, again, the advantage of arthroscopic surgery for the preservation of involved joint function, particularly that of knee joints needs to be considered.

**Table 5:** Recent studies associated with the results of the present study.

Authors	Cohort	Recurrence	Risk factors for recurrence
Mastboom et al.	966 cases surgically treated	425(44%) RFS(3 years): 62% RFS(5 years): 55%	Knee (P = 0.10) Arthroscopic surgery (P = 0.03) Recurrent disease (P < 0.0001)
Patel et al.	214 cases (knee)	Localized: 8.6% Diffuse: 47.6%	Arthroscopic surgery (Diffuse, P = 0.0004)
Keyhani et al.	21 cases (Knee, diffuse)	No recurrence (All arthroscopic surgery) (clinical)	N.A.
Vespoor et al.	206 cases	Localized: 6% Diffuse: 28%	N.A. Health related QOL: good (Pain scores: varies among patients)

(i) Mastboom^30^.

(ii) Patel^26^.

(iii) Keyhani^31^.

(iv) Verspoor^32^.

Few studies have mentioned or investigated the progression of osteochondral destruction after surgery. Multivariate analysis showed osteochondral destruction at the initial examination and local recurrence to be independent risk factors for the progression of osteochondral destruction in the present study. Local recurrence itself sometimes causes pain and limitation of range of motion, while osteochondral destruction often impairs the patient QOL. Although the local recurrence rate was higher in cases with arthroscopic surgery compared with open synovectomy, osteochondral destruction was attributable to arthroscopic surgery in only a single case, although the follow-up period was short. The results of our previous report indicated that repeated surgery for local recurrence was a significant risk factor for progression of osteochondral destruction^21^. However, the results of the present study did not show repeated surgery to be a risk factor for osteochondral destruction. This difference between the previous and present studies is related to the transition over time of the treatment procedure for TSGCT of the anterior knee from open total synovectomy to arthroscopic surgery.

Sharma et al.^29^ reported that repeated surgery could salvage relapses, but was associated with morbidity after additional surgeries. They also cited Chin et al.’s study in which complications such as stiffness, contractures, and reflex sympathetic dystrophy were noted more frequently with open procedures^13^. Because TSGCT is a benign tumor, invasive treatment is not always needed to only reduce the local recurrence rate. Although recurrence and its effects on the subsequent clinical features after arthroscopic surgery should be further analyzed in the future, this procedure, particularly for knee location, may be an appropriate technique to reduce osteochondral destruction.

Other than knee joints, hip joints have also previously been reported to be destroyed by TSGCT^11^ and the results of the present study agreed. A previous study reported the underlying mechanism of osteochondral destruction in diffuse type TSGCT. Namely, TSGCT invades bone from the attachment site of the articular capsule or ligaments where articular cartilage is bare^20^. Furthermore, previous studies including our previous report indicated that osteochondral destruction often occurs in joints with a small articular cavity such as hip and ankle^12,15,21^. These previous results are consistent with the findings of the current study in which diffuse-type and joints other than knee were independent factors associated with the initial joint destruction. Diffuse type TSGCT could invade bone at areas bare of cartilage, with joints other than knees having low joint capacity. In these joints, reconstruction procedures for joints including total joint arthroplasty and arthrodesis are appropriately selected depending on the individual circumstances.

Biomarkers that can predict the prognosis will aid in the understanding of this tumor. Since CSF1 overexpression is associated with the pathology of TSGCT, two studies have reported an association between CSF1 expression and pathological subtype (localized or diffuse type), and/or an association between CSF1 expression and local recurrence or bone destruction^33,34^. CSF1 expression was determined with immunohistochemical staining for CSF1 in 26 cases with pathological tissues available. The cutoff value for positive CSF1 stainability was determined according to a previous report^33^. The local recurrence survival was lower in the CSF1 high expression group compared with low expression group, but no significant difference was observed (P = 0.32). In addition, there was no significant difference between CSF1 expression and osteochondral destruction at the first visit (P = 0.59) or progression of osteochondral destruction (P = 0.60). Whether biomarkers containing CSF1 expression predict the prognosis of TSGCT remains to be determined. Analysis with an increased number of cases will be needed in the future.

Several limitations are present in the present study. First, it focused on a limited number of patients due to the rarity of this disease, who were analyzed retrospectively. Exclusion of cases followed up for less than 12 months and with insufficient medical records reduced the number of localized cases such as those with finger or toe involvement. Because the present study focused on the factors associated with local recurrence and osteochondral destruction causing possible functional impairment, cases with relatively large joints were selected as targets for investigation. Compared with previous reports, the present eighty cases represent a relatively large cohort, and analyses for osteochondral destruction have rarely been performed. In addition, the limited number of patients particularly other than knee cases made it difficult to perform statistical analyses. It will be necessary to conduct a national or international study to facilitate better statistical analysis of joints other than the knee. The second limitation is that numerical evaluations of patients’ function after surgery, local recurrence, and osteochondral destruction were not performed, and the occurrence of osteochondral destruction is not necessarily consistent with the onset of pain. In future studies, function in addition to the presence of pain should be analyzed for not only surgically treated patients, but also those treated conservatively including watchful observation. The third limitation was the presence of slight differences in treatment strategies among the treatment periods and institutions. The final treatment modality was determined by the physicians and patients based on close counseling at each institution, and in some institutions full-time orthopedic surgeons specializing in arthroscopic surgery are not available. Indications for specific surgical procedure, arthroscopic surgery or open surgery, differ among cases. These factors may have influenced the outcome. Although the efficacy of radiotherapy for TSGCT has been reported^35,36^, it has in general not been chosen in this context at our institutions, or in our country. Another limitation is that it would be necessary to evaluate by MRI instead of plain radiography or CT in order to evaluate cartilage damage more accurately.

## Conclusions

Considering that the purposes of treatment for TSGCT are to reduce local recurrence and maintain the functions of involved joints, it is important for physicians to know the factors influencing local recurrence and progression of osteochondral destruction. Diffuse type is a factor that should be noted for both local recurrence and osteochondral destruction, and local recurrence occurs but osteochondral destruction is less observed in the knee.

## Data Availability

The datasets generated during and/or analyzed during the current study are available from the corresponding author on reasonable request.

## Abbreviations

TSGCT: Tenosynovial giant cell tumor
PVNS: Pigmented villonodular synovitis
MRI: Magnetic resonance imaging
CT: Computed tomography
K–L: Kellgren–Lawrence
LRFS: Local recurrence free survival rate
CSF: Colony stimulating factor
RANKL: Receptor activator of nuclear factor-kappa B
